# Biological effects of the oxygen molecule in critically ill patients

**DOI:** 10.1186/s40560-020-00505-9

**Published:** 2020-12-14

**Authors:** Masaki Nakane

**Affiliations:** Department of Emergency and Critical Care Medicine, Yamagata University Hospital, 2-2-2 Iida-nishi, Yamagata, 990-9585 Japan

**Keywords:** Hypoxia, Hyperoxia, Oxygen, Oxidative stress, Reactive oxygen species, Lactate, Critical care

## Abstract

The medical use of oxygen has been widely and frequently proposed for patients, especially those under critical care; however, its benefit and drawbacks remain controversial for certain conditions. The induction of oxygen therapy is commonly considered for either treating or preventing hypoxia. Therefore, the concept of different types of hypoxia should be understood, particularly in terms of their mechanism, as the effect of oxygen therapy principally varies by the physiological characteristics of hypoxia. Oxygen molecules must be constantly delivered to all cells throughout the human body and utilized effectively in the process of mitochondrial oxidative phosphorylation, which is necessary for generating energy through the formation of adenosine triphosphate. If the oxygen availability at the cellular level is inadequate for sustaining the metabolism, the condition of hypoxia which is characterized as heterogeneity in tissue oxygen tension may develop, which is called dysoxia, a more physiological concept that is related to hypoxia. In such hypoxic patients, repetitive measurements of the lactate level in blood are generally recommended in order to select the adequate therapeutic strategy targeting a reduction in lactate production. Excessive oxygen, however, may actually induce a hyperoxic condition which thus can lead to harmful oxidative stress by increasing the production of reactive oxygen species, possibly resulting in cellular dysfunction or death. In contrast, the human body has several oxygen-sensing mechanisms for preventing both hypoxia and hyperoxia that are employed to ensure a proper balance between the oxygen supply and demand and prevent organs and cells from suffering hyperoxia-induced oxidative stress. Thus, while the concept of hyperoxia is known to have possible adverse effects on the lung, the heart, the brain, or other organs in various pathological conditions of critically ill patients, and no obvious evidence has yet been proposed to totally support liberal oxygen supplementation in any subset of critically ill patients, relatively conservative oxygen therapy with cautious monitoring appears to be safe and may improve the outcome by preventing harmful oxidative stress resulting from excessive oxygen administration. Given the biological effects of oxygen molecules, although the optimal target levels remain controversial, unnecessary oxygen administration should be avoided, and exposure to hyperoxemia should be minimized in critically ill patients.

## Introduction

Supplemental oxygen therapy has been long recognized as a common treatment administered in the hospital setting, both in the acute and chronic phases, and is widely applied throughout treatment, from pre-hospital emergency medical services to home oxygen therapy. It is extremely important to remember that oxygen is a prescription drug with specific biochemical and physiological actions, an adequate range of effective doses and well-known adverse effects at excessively high doses [1].

The oxygen molecule was first detected early in the 1770s and was recognized as a chemical element by correctly characterizing its role in combustion and corrosion; it received the proper name ‘oxygen’ in 1777. Interestingly, oxygen is the most abundant chemical element by mass in the Earth’s biosphere, including the air, sea, and land. In addition, oxygen gas is recognized as the second-most common component of the Earth’s atmosphere, accounting for 20.8% of its volume and 23.1% of its mass. Supplemental oxygen as a medical treatment became common early in the twentieth century for patients who suffered discomfort or difficulty breathing, mainly helping them recover from a hypoxic state and relieve dyspnea. When the indications for oxygen therapy are considered, hypoxia, or hypoxemia is the most important pathophysiological state which needs to be appropriately treated and fully understood by medical healthcare providers.

## Hypoxia and hypoxemia

Hypoxia is generally defined as a condition in which the whole body (systemic hypoxia) or a region of the body (regional hypoxia) is deprived of adequate oxygen supply at the tissue level, including disorder of oxygen utilization, and differs from hypoxemia, which refers specifically to a condition of low oxygen tension in arterial blood. Hypoxia has been medically classified into five different pathophysiological conditions: atmospheric hypoxia, hypoxemic/hypoxic hypoxia, anemic hypoxia, circulatory/hypoperfusion/ischemic hypoxia and toxic/histotoxic hypoxia [2, 3]. A further classification from the perspective of the causes of hypoxia is described as follows:

1. Inadequate oxygenation of the blood in the lungs due to extrinsic reasons, such as deficiency of oxygen in the atmosphere (e.g., high altitude) or hypoventilation caused by neuromuscular and/or respiratory center disorders.
2. Pulmonary diseases that can lead to hypoventilation caused by increased airway resistance or decreased pulmonary compliance, an abnormal ventilation-perfusion ratio or diminished respiratory membrane diffusion.
3. Venous-to-arterial shunts (right-to-left cardiac shunts)
4. Inadequate oxygen transport to the tissues by the blood due to anemia or abnormal hemoglobin (e.g., carboxyhemoglobin, methemoglobin), systemic circulatory failure, localized circulatory failure, or tissue edema.
5. Inadequate oxygen utilization in tissue caused by poisoning of cellular oxidation enzymes or a diminished cellular metabolic capacity for using oxygen caused by toxicity (e.g., cyanide poisoning), vitamin B_1_ deficiency (e.g., beriberi), or other factors.

It is important for clinicians and nurses to understand the concept of different types of hypoxia, as the effects of oxygen therapy vary by the physiologic characteristics of each hypoxia type [3]. For example, oxygen therapy is of much less value in cases of hypoxia caused by anemia or abnormal hemoglobin, circulatory failure or physiological shunt than in other situations because a normal level of oxygen already exists and is available in the alveoli, with hypoxia rooted in other causes. However, oxygen therapy can be extremely beneficial for treating atmospheric hypoxia by correcting the depressed oxygen level in the inspired gases and it is also markedly effective for treating hypoventilation hypoxia by facilitating the inspiration of more oxygen into the alveoli on each breath than with normal air. In cases of hypoventilation hypoxia, however, oxygen therapy provides no beneficial effects for resolving the simultaneous carbon dioxide retention often caused by hypoventilation.

### Oxygen sensing in hypoxia

**Fig. 1:**
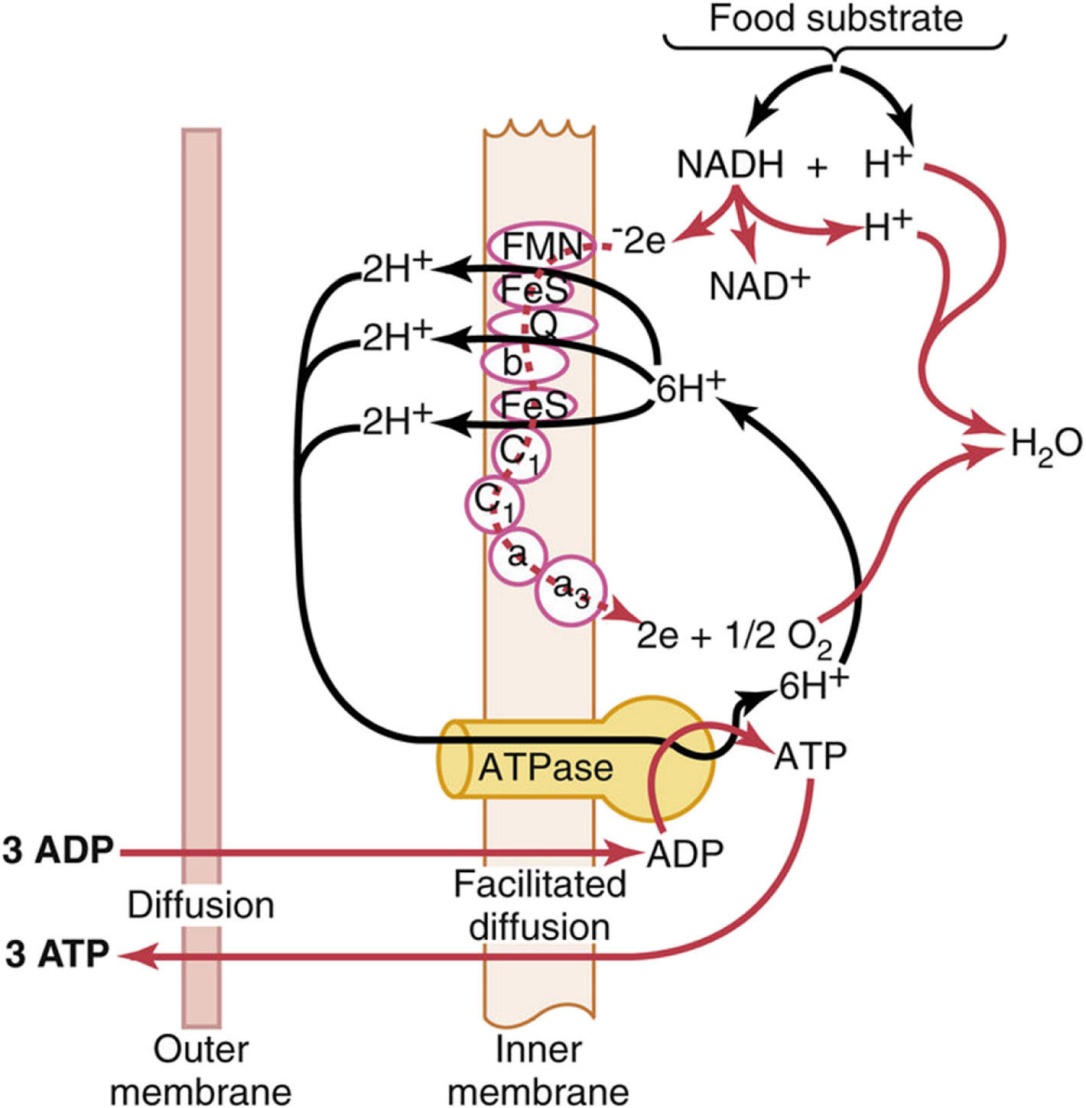
Chemiosmotic mechanism of oxidative phosphorylation in mitochondria to form adenosine triphosphate (ATP). ADP: adenosine diphosphate, FeS: iron sulfide protein, FMN: flavin mononucleotide, NAD^+^: nicotinamide adenine dinucleotide, NADH: reduced nicotinamide adenine dinucleotide, Q: ubiquinone. (Reuse from Guyton and Hall Textbook of Medical Physiology 14th ed. with permission from Elsevier B.V.)

Oxygen must be constantly supplied to all cells of the human body for their survival and for use as the final electron acceptor in the process of oxidative phosphorylation, which involves the oxidation of hydrogen through a series of enzymatically catalyzed reactions in mitochondria and is necessary to release energy to form adenosine triphosphate (ATP) (Fig. 1). Therefore, both systemic and cellular oxygen-sensing systems based on the mechanisms underlying oxygen homeostasis exist to tightly regulate oxygen levels within cells and tissues of the whole body in order to protect the human body from hypoxia.

As is well known, the respiratory center is located in the medulla oblongata and pons of the brain stem, which plays an important physiological role in regulating the respiratory drive. However, changes in the oxygen concentration have no direct effect on the respiratory center itself, although they do have an indirect effect acting through the peripheral chemoreceptors. These chemoreceptors, most of which are located in the carotid bodies in the bilateral carotid arteries and a few in the aortic bodies along the aortic arch, are especially important for detecting changes in the oxygen concentration in the arterial blood. Once the oxygen concentration in the arterial blood falls below normal, the chemoreceptors become strongly stimulated and act as a systemic oxygen-sensing system, sending signals to the respiratory center to promote the respiration function.

Hypoxia-inducible factors (HIFs), described as critical for the survival of all metazoan species as master regulators of oxygen homeostasis [4, 5], are heterodimeric proteins consisting of an oxygen-regulated HIF-1α, HIF-2α, or HIF-3α subunit and a HIF-1β subunit [6], which exist in all nucleated cells. Depending on the context, both HIF-1 and HIF-2, essential for sensing oxygen via the carotid bodies, activate the transcription of numerous target genes that mediate adaptive responses, including erythropoiesis, vascularization (angiogenesis), metabolic reprogramming and ventilatory acclimatization induced by continuous hypoxia in order to regulate oxygen delivery at the systemic level and to regulate oxygen utilization at the cellular level [4, 7–11], functioning as a cellular oxygen-sensing system. In addition to the various adaptive responses, maladaptive (pathophysiological) responses, including cardiovascular pathology, can be evoked due to persistent oxidative stress induced by chronic intermittent hypoxia associated with recurrent apnea in sleep-disordered breathing; these responses are reported to also be mediated by HIF-1 and HIF-2 in the carotid bodies [4, 7–11].

Both these systemic and cellular oxygen-sensing mechanisms against hypoxia are employed to ensure a proper balance between the oxygen supply and demand, respectively. Dysregulation of these mechanisms results in promoting disease pathology.

### Dysoxia beyond hypoxia

Under critically ill conditions, such as sepsis, shock and cardiac arrest/resuscitation, the signs of hypoxia and reduced oxygen extraction have been reported to persist despite the recovery of systemic oxygen delivery. Circulatory compromise resulting in an inability to maintain adequate oxygen transport to peripheral cells has been attributed not to systemic alterations but to the failure of microcirculation and mitochondria to transport and efficiently use oxygen [12]. The strategy of targeting supra-normal levels of systemic oxygen delivery impressively proposed by Shoemaker et al. [13] in the 1990s based on the notion that a relative shortage of delivered oxygen causes a deficit in tissue oxygenation was not found to be a valid strategy based on the findings of several clinical trials showing no marked differences in mortality or morbidity in patients with sepsis compared with that of targeting normal levels of systemic oxygen delivery. As an underlying reason for this fact, the deficit in oxygen extraction was thus proposed to not be caused by insufficient systemic oxygen delivery, but rather by pathological heterogeneity of microvascular perfusion, thus resulting in the functional shunting of the microcirculation which clinically manifests as a reduction in oxygen extraction [14, 15].

The heterogeneity in tissue oxygen pressures between physiologically different compartments has become central to heated debates, ultimately resulting in the new definition of a more physiological concept related to hypoxia, termed “dysoxia.” Dysoxia is a condition of hypoxia wherein the oxygen availability at the cellular level is inadequate to sustain enough oxidative phosphorylation to form ATP [15–17]. Oxygen transport and utilization are thus considered to be highly dependent on the functional morphology and metabolism of each organ, underlying disease state, duration of disease, and type of therapy administered [18].

### Lactate production during hypoxia

Recently, the blood lactate concentration has been widely measured in patients with sepsis, shock, post-cardiac arrest, or a hypermetabolic state, as well as in those with glucose catabolism (mostly diabetic patients). However, lactate is not just a metabolic waste product generated by anaerobic glycolysis after switching from aerobic metabolism in a hypoxic state. Even under normal conditions, marked levels of lactate are produced in the human body, at as high a rate as approximately 1.5 mol/day, and its role in the distribution of oxidative and gluconeogenic substrates as well as cell signaling has been highlighted [19, 20] and described as the “lactate shuttle theory” [21].

Glycolysis metabolizes glucose to pyruvate, which then proceeds to 1 of 2 routes for further metabolism: either the aerobic pathway, which can effectively generate 38 ATP molecules with oxidative phosphorylation via the citric acid cycle (i.e., Krebs cycle) from each molecule of metabolized glucose, or the anaerobic pathway, which leads to massive lactate production due to bilateral catalysis by lactate dehydrogenase with a normal lactate-to-pyruvate ratio of approximately 10:1.

**Fig. 2:**
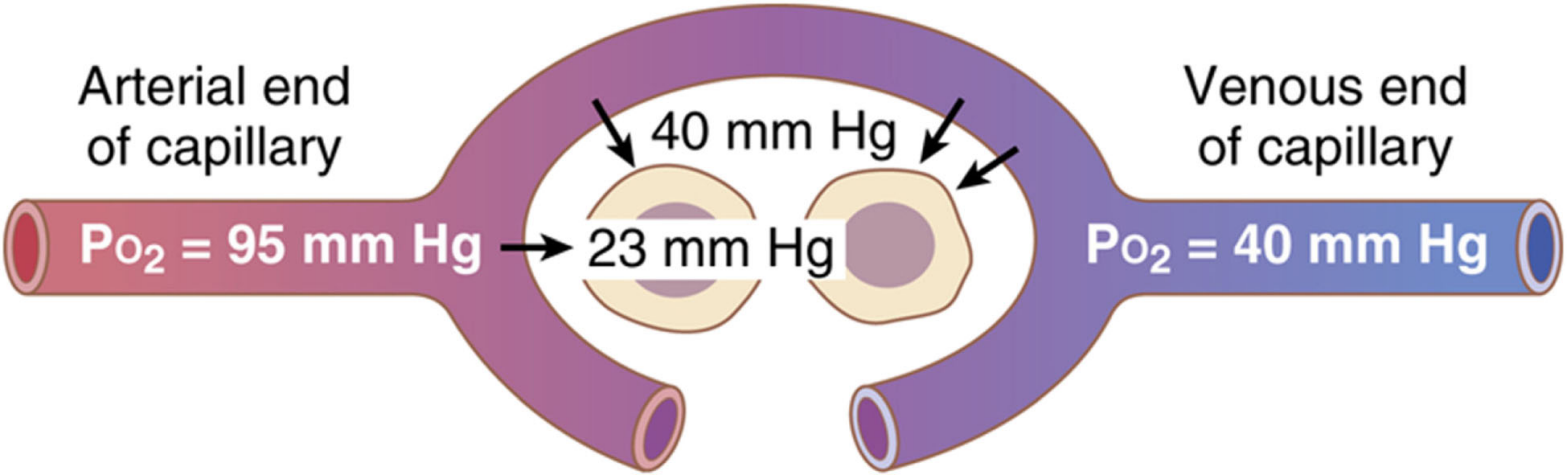
Diffusion of an oxygen molecule from the arterial end of a capillary to tissue cells. PO_2_ in the interstitial fluid averaged 40 mmHg, and that in the cells was 23 mmHg, ranging from 5 to 40 mmHg. (Reuse from Guyton and Hall Textbook of Medical Physiology 14th ed. with permission from Elsevier B.V.)

When sufficient oxygen cannot be utilized, i.e., hypoxia, the citric acid cycle stops metabolizing pyruvate to obtain ATP, instead progressively generating lactate. At this point, one could ask how hypoxic state leads to switching aerobic to anaerobic pathway. When the arterial blood reaches the peripheral tissues, the partial pressure of oxygen (PO_2_) in the capillaries is still around 95 mmHg. As shown in Fig. 2, the PO_2_ in the interstitial fluid that surrounds the tissue cells averages only 40 mmHg, and the intracellular PO_2_ further decreases to an average 23 mmHg, ranging between 5 and 40 mmHg by diffusion of oxygen molecules into cells. Thus, there is a large initial pressure difference that causes oxygen to diffuse rapidly from the capillary blood into the tissues and then gradually into the cells.

**Fig. 3:**
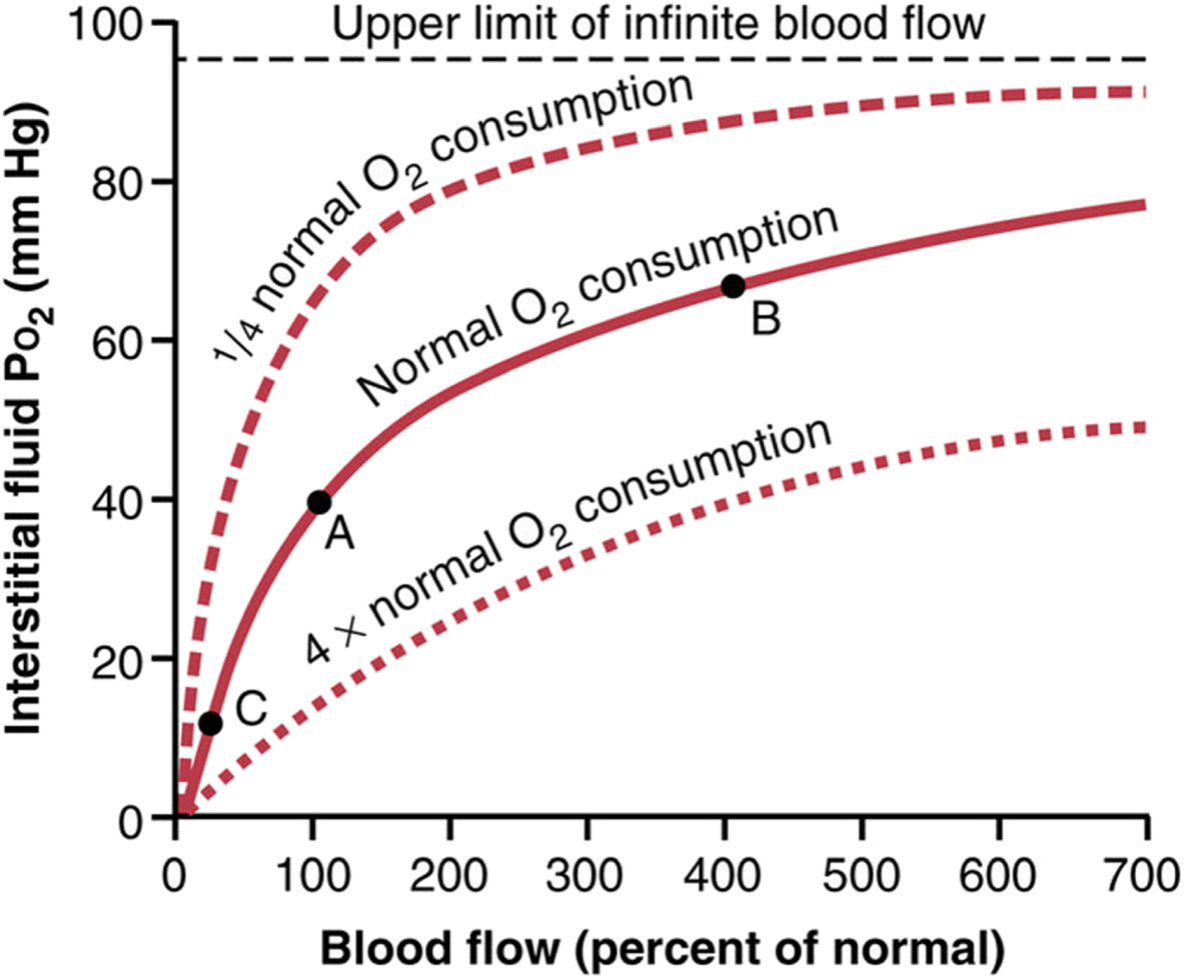
Effect of the blood flow and rate of oxygen consumption on tissue PO_2_. An increase in blood flow to 400% from point A to B increases the tissue PO_2_ from 40 to 66 mmHg, limited to 95 mmHg, just as with the arterial PO_2_, even with maximal blood flow. Conversely, if the blood flow decreases from point A to C, the tissue PO_2_ also decreases. (Reuse from Guyton and Hall Textbook of Medical Physiology 14th ed. with permission from Elsevier B.V.)

**Fig. 4:**
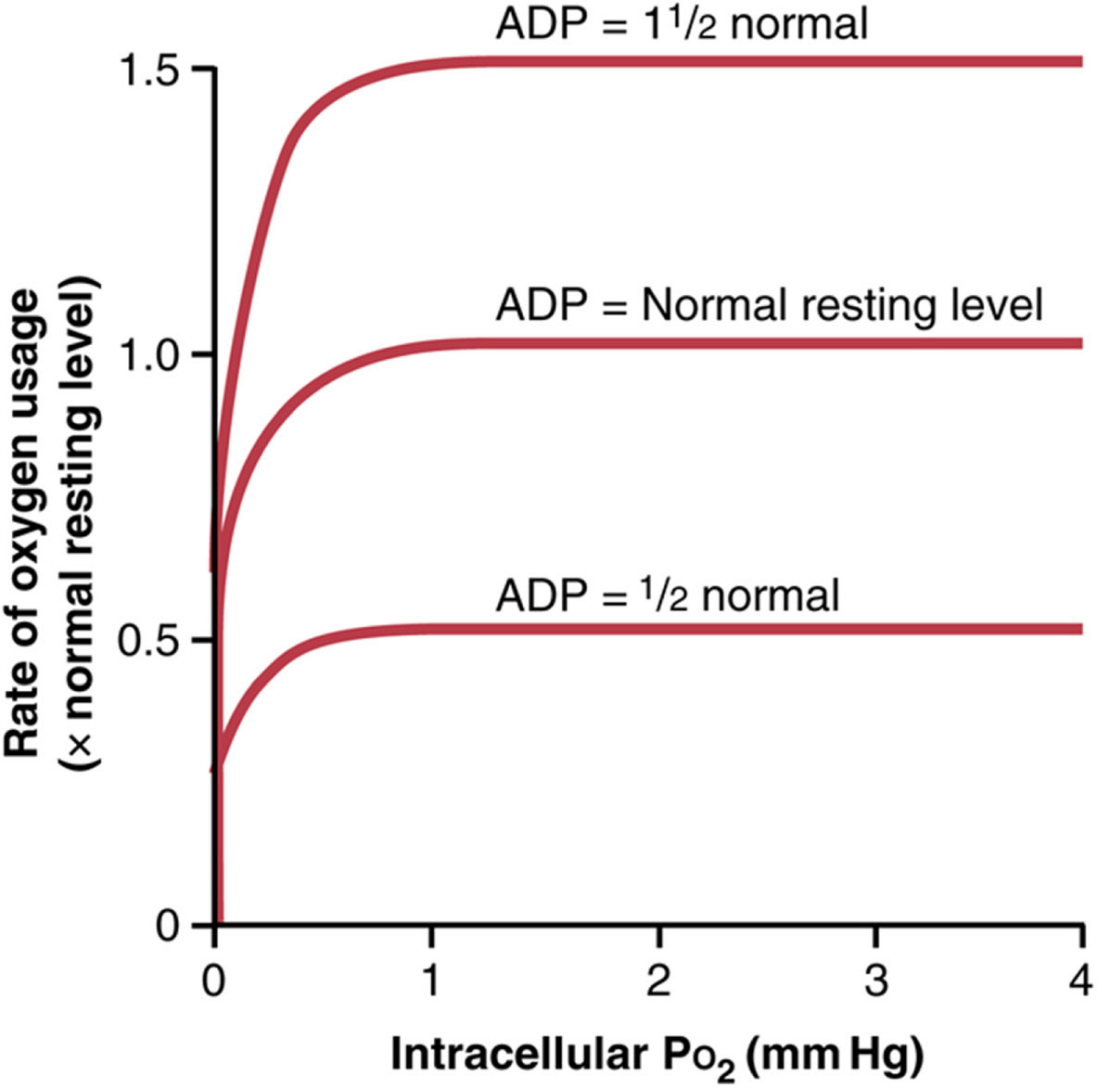
Relationship between PO_2_ and the rate of oxygen usage by the cells at different concentrations of intracellular adenosine diphosphate (ADP). As long as intracellular PO_2_ remains above 1 mmHg, the rate of oxygen usage remains constant, depending on the ADP concentration. (Reuse from Guyton and Hall Textbook of Medical Physiology 14th ed. with permission from Elsevier B.V.)

Interstitial fluid PO_2_ is theoretically reduced by both decreases in the blood flow in the capillary and increases in the cellular oxygen consumption of the tissues (Fig. 3). In brief, tissue PO_2_ is determined by a balance between the rate of oxygen transport to the tissues and the rate at which the oxygen is used by the tissues. However, the metabolic use of oxygen by cells to produce ATP with oxidative phosphorylation will theoretically remain constant until the intracellular PO_2_ decreases to a level of 1 mmHg (Fig. 4). Progressive lactate production would thus not occur until extremely hypoxic conditions (< 1 mmHg of PO_2_) appeared. Therefore, the disturbance of the cellular normal function leading to hyperlactatemia in critically ill patients cannot be attributed solely to issues with oxygen transport.

However, hyperlactatemia can also occur under specific conditions independent of hypoxia. The entry of pyruvate into the citric acid cycle can be limited by a deficiency of thiamin (vitamin B_1_) which plays an important role and it is a coenzyme that is required for the catalytic activity of several enzymes, including pyruvate dehydrogenase, which converts lactate to pyruvate, and thus diverts pyruvate production to lactate production.

In patients with sepsis and septic shock, an increase of “glycolytic flux” exceeding the oxidative capacity of mitochondria can occur and result in increased pyruvate production and hence lactate production. Since hyperlactatemia is reported to be a powerful indicator with a strong correlation with the severity of sepsis and is caused more frequently by impaired tissue oxygen use than by impaired oxygen transport [22], it is important to select an adequate therapeutic strategy carefully targeting a decrease in lactate production as a reliable marker [23]. Interestingly, lactate production, possibly via glycolytic flux, can be reduced by β2-antagonists such as esmolol in septic patients despite reduced oxygen delivery [24] and by sodium-potassium-adenosine triphosphatase inhibitors in microdialized fluid obtained from the quadriceps muscles of septic patients [25] and in the animal skeletal muscle of induced glycolysis by epinephrine [26, 27]. Thiamin is also expected to exert some beneficial effects on septic patients and is thus considered a component of combination treatment with hydrocortisone, ascorbic acid (vitamin C), and thiamin (HAT therapy) [28]. However, a recent prospective randomized clinical trial [29] evaluating the efficacy and safety of HAT therapy for patients with sepsis and septic shock unfortunately failed to show a significant reduction in mortality and no marked improvement in any other secondary outcomes, which is consistent with the results of the retrospective study by Litwak et al. [30]. The combined treatment of such components could not fundamentally resolve cellular hypoxia in patients with sepsis, probably because it is not just a simple case of oxygen shortage.

## Hyperoxia and hyperoxemia

Hyperoxia is basically an excess of oxygen in the body tissues compared to normoxia and most commonly occurs in patients breathing supplemental oxygen to minimize tissue hypoxia. In subjects with a normal respiratory function, the administration of any fraction of inspired oxygen (F_I_O_2_) higher than 0.21 will lead to hyperoxemia (i.e., arterial hyperoxia), a condition in which the arterial PO_2_ (PaO_2_) rises above the normal range of 80–100 mmHg, which is generally used to define normoxemia in a subject breathing room air [31]. However, various criteria have been used in clinical studies to define hyperoxemia, leading to highly inconsistent results [32]. In most observational studies, patients were categorized as either hyperoxemic or non-hyperoxemic based on arbitrarily predetermined PaO_2_ cut-off values, ranging from as high as 300 mmHg to less extreme as 200 or 120 mmHg [32, 33].

### Oxygen sensing in hyperoxia

**Fig. 5:**
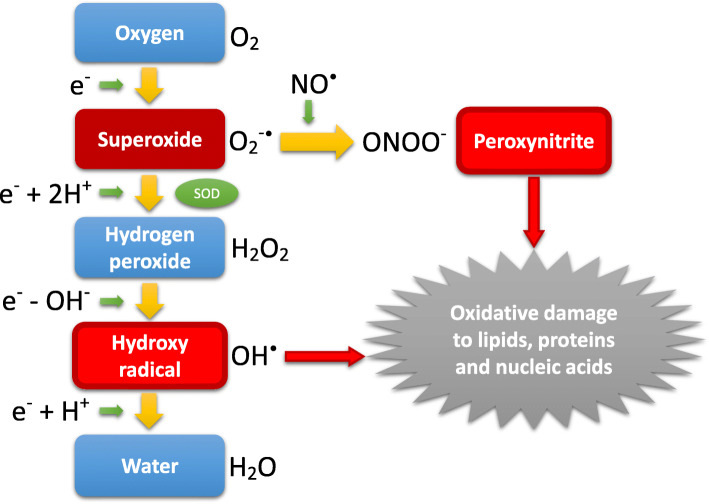
In the process of the stepwise reduction from oxygen to water, reactive oxygen species (ROS) are produced. Both hydroxy radical and peroxynitrite are the most reactive agents among the species

Sustained deviation over normoxia, which is defined as the level of oxygen required for normal physiological processes to occur, leads to the increased production of reactive oxygen species (ROS) (Fig. 5), which can cause the oxidation of lipids, proteins, and nucleic acids, possibly resulting in cellular dysfunction or death [34]. In contrast to hypoxia, however, humans have not evolved any specific adaptation to counter hyperoxia, so there exists no systemic mechanism to elegantly handle unusual instances of hyperoxia. Despite this, cellular responses to hyperoxia have been reported to occur along with signaling initiated by the patient’s underlying pathology [35].

Since the lungs are directly exposed by hyperoxia, which can cause inflammatory lung injury and compromise the innate immunity, pulmonary oxygen toxicity has been well studied, which has led to the definition of hyperoxia-induced acute lung injury (HALI), characterized by inflammatory responses with leukocyte infiltration, injury, and death of epithelia, endothelia, and macrophages. In addition to directly modifying macromolecules, exposure to hyperoxia involves the activation of ROS, which mediates the direct and indirect modulation of many hyperoxia-sensing molecules, such as protein kinase (i.e., MAPK, PKC, PI3-kinase, Akt), redox-sensing transcription factors (i.e., NF-E2, Nrf2, NF-kB, AP-1, CREB), receptors (i.e., CXCR2, RAGE, TRL), and pro-/anti-apoptotic factors [35]. Therefore, a clearer understanding of hyperoxia-induced signal transduction pathways that contribute to the pathophysiological progress of HALI is crucial to facilitate the design of successful therapeutic strategies as well as prevention strategies [35].

### Harmful effects of hyperoxia and hyperoxemia

When the PaO_2_ was analyzed as a continuous variable, a linear relationship between increasing O_2_ tension and mortality was found, without a clear threshold for harm [36, 37]. A recent cohort study showed that the incidence of hyperoxemia in the intensive care unit (ICU), as well as the strength of the association with a worse outcome, varied markedly depending on the particular metric applied, with metrics of central tendencies (mean and median PaO_2_) showing the strongest relationship with the outcome [38]. Although severe hyperoxemia (PaO_2_ > 200 mmHg) was more consistently associated with an adverse outcome than the other risk factors, mortality appeared to increase linearly with the exposure time even in cases of mild hyperoxemia (PaO_2_ of 120–200 mmHg) [38].

While hyperoxia and hyperoxemia have been defined in various manners [39], as also explained above, there seems to be several potential adverse events commonly seen in patients who receive supplemental oxygen (Table 1), summarized as physical effects, physiological effects, biochemical and cellular effects [39, 40].

**Table 1:** Characteristics of the biological effects of oxygen molecules

✓ Oxygenation	
• Increase in arterial oxygen content and hence systemic oxygen delivery	
• Variable efficacy depending on the type of hypoxia	
✓ Circulation	
• Systemic vasoconstriction	
• Increase in systemic vascular resistance and hence systemic arterial pressure	
• Decrease in cardiac stroke volume and cardiac output	
• Possible decrease in tissue blood flow	
• Pulmonary vasodilation in affected area by inhaled oxygen	
• Decrease in pulmonary vascular resistance and hence pulmonary arterial pressure	
• Increase in pulmonary blood flow	
• Decrease in right-to-left intracardiac shunt flow, if it exists	
• Regional perfusion	
• Decrease in coronary blood flow	
• Aggravation of ischemic damage in myocardial infarction	
✓ Cellular toxicity	
• Inflammation: Induction of pro-inflammatory cytokines	
• Production of reactive oxygen species	
• Enhanced neurological damage after ischemia reperfusion	
• Induced lung injury and possible dysfunction of other organs	
✓ Others	
• Occurrence of resorption atelectasis	
• Impairment of mucociliary clearance in trachea/bronchus	
• Compromised innate immunity	

Physical effects include adverse events as drying and cooling of the airway, especially when the oxygen is administered at a relatively high flow rate, leading to not only the patient’s discomfort but also adverse effects on the respiratory mucous blanket and mucociliary clearance of sputum and secretions from the trachea and bronchus, which may lead to sudden airway obstruction due to dried sputum in the worst case. Recently, increased interest has been focused on nasal high-flow therapy, which uses a 20–60-L/min gas flow of a mixture of oxygen, enabling the administered gas to be warmed and humidified enough to prevent such adverse events [41, 42].

The physiological effects induced by supplemental oxygen include changes in the hemodynamics such as vasoconstriction of the systemic circulation and vasodilation of the pulmonary vasculature. However, these phenomena would likely have little effect on the systemic condition in critically ill patients except for patients who have congenital heart disease complicated with intracardiac shunt or a postoperative status with Fontan circulation, wherein the pulmonary blood flow is easily affected by pulmonary vascular resistance. In addition to that, it is important to elucidate how the oxygen levels may affect to the inflammatory response. High oxygen concentrations may indirectly ameliorate the inflammatory response by reducing the degree of tissue hypoxia and, as a consequence, the levels of HIF [4–11], a key regulatory molecule of both hypoxia and the inflammatory response [43]. More importantly in clinical situations, if patients with chronic hypercapnia, such as moderate to severe chronic obstructive pulmonary disease (COPD) or neuromuscular disease with ventilatory failure, are given uncontrolled excessive oxygen therapy, they may develop worsening hypercapnic (type II) respiratory failure and suffer severe respiratory acidosis, falling unconscious [44]. It should also be noted that resorption atelectasis frequently occurs after breathing high oxygen concentrations, even in normal subjects, by removing nitrogen from the alveoli and thus causing the more rapid diffusion of inspired oxygen into the blood, leading to alveolar collapse [45–48].

Biochemical and cellular effects are prominently represented by oxygen toxicity, a condition that results from the harmful effects of breathing oxygen at elevated partial pressures. The mechanisms underlying oxygen toxicity can primarily be explained by the formation of ROS including oxygen free radicals and biochemically based on the effects of hyperoxia. Oxygen free radicals have one or more unpaired electrons, which makes them very unstable. The most biologically significant agents among these ROS are hydroxyl radical (OH^•^) and peroxynitrite (ONOO^−^) (Fig. 5), both of which are oxygen free radicals. Peroxynitrite, the product of the reaction between superoxide anion (O_2_^−•^) and nitric oxide (NO), interacts with lipids, proteins, and nucleic acids directly through oxidative stress [34] or indirectly through radical-mediated mechanisms [49]. These reactions trigger cellular responses ranging from the subtle modulation of cellular signaling to overwhelming oxidative stress that can induce cells to undergo necrosis or apoptosis [49]. The understanding of the molecular mechanisms underlying both hypoxia and hyperoxia is still developing [35, 43, 49, 50].

## Clinical implications of oxidative stress

Breathing with excess oxygen may induce oxidative stress as an unavoidable consequence [51] by initiating the production of oxygen free radicals, as previously mentioned. Mitochondria have a variety of anti-oxidant systems that promote the detoxification of ROS generated during aerobic metabolism [34, 52]. Superoxide dismutase (SOD), a family of metalloenzymes, exerts an anti-oxidant effect by enzymatically converting superoxide anion to hydrogen peroxide (H_2_O_2_) (Fig. 5). The expression of SOD is further induced by oxidative stress as hyperoxia in a process mediated by the oxidative activation of the nuclear transcription factor NFκB [34]. Since superoxide anion can be a precursor of both hydroxy radical via H_2_O_2_ and peroxynitrite (Fig. 5), it is important to keep its steady-state concentration at the lowest possible level by utilizing such variable mechanisms as different SOD isozymes, reduction of cytochrome c, and lowering the pH [34]. Other anti-oxidant agents that eliminate ROS include catalase and glutathione peroxidase against H_2_O_2_, ubiquinol and coenzyme Q (acting as a reducing agent), vitamin E (interfering with free radical-mediated chain reaction), cytochrome c oxidase (acting as a peroxidase) [34, 52], glutathione, and bilirubin [52]. In addition to the anti-oxidant defenses mentioned above, mitochondria have a variety of DNA-repairing enzymes to correct errors resulting from oxidative damage [34].

A critically ill status with systemic inflammation and shock may provoke an overproduction of ROS that overwhelms the anti-oxidant system of the human body. In such situations, the administration of excess oxygen may contribute to an undesirable phenomenon (Table 1) associated with the imbalance between pro- and anti-oxidant agents and consequently aggravate oxidative stress, damaging lipids, proteins and nucleic acids and leading to potential cell death, apoptosis [49], and the exacerbation of the inflammatory response [52].

Based on the concept that liberal use of oxygen leads to unexpected hyperoxia, which may increase harmful oxidative stress, thereby resulting in worse clinical outcomes, some randomized controlled trials have been recently conducted to compare conservative oxygen therapy with usual oxygen therapy [53, 54]. The multicenter, bi-national ICU-ROX (ICU randomized trial comparing two approaches to oxygen therapy) showed that the use of conservative oxygen therapy did not significantly affect the number of ventilator-free days (21.3 vs. 22.1 days) or the 90- and 180-day mortality (34.7% vs. 32.5% and 35.7% vs. 34.5%) in adult patients who were expected to undergo mechanical ventilation in the ICU beyond the day after study recruitment [54]. In addition, early exposure to a conservative oxygenation strategy (target PaO_2_ 55–70 mmHg) for 7 days did not reduce the 28-day mortality, the ICU mortality and the 90-day mortality (34.3% vs. 26.5%, 36.4% vs. 26.5% and 44.4% vs. 30.4%) compared with liberal oxygen therapy (target PaO_2_ 90–105 mmHg) in mechanically ventilated patients with acute respiratory distress syndrome [55].

### Systemic responses

Enhanced oxidative stress may have also a systemic impact on hyperoxia. In an animal model of sepsis, hyperoxemia for 24 h was associated with elevated serum levels of ROS and inflammatory cytokines, an increased spread of infection and worsening multiple organ dysfunction [56]. From a hemodynamic perspective, hyperoxia induces systemic vasoconstriction, which increases systemic vascular resistance with consequent reductions in the heart rate, stroke index, and cardiac index [57], while the inspiration of high-concentration oxygen causes pulmonary vasodilation, which directly decreases pulmonary vascular resistance. The oxidative stress-induced reduction in the bioavailability of NO, a crucial intrinsic vasodilator, seems primarily responsible for systemic vasoconstriction [58]. Furthermore, a recent study using isolated microvascular endothelial cells also showed a decrease in the cell viability and proliferation under hyperoxic conditions [59]. Hyperoxia reportedly impaired microvascular perfusion [60–62] and induced a paradoxical reduction in regional oxygen delivery [63] in various clinical situations, although other experimental reports have suggested a beneficial role of hyperoxemia in hemodynamic stabilization and the redistribution of blood flow to important organs [64, 65].

### Effects on the lung

When excessive oxygen is administered to patients, the lung is the first organ involved. Exposure to 100% oxygen was reported to cause oxidative stress and inflammation in animal lungs, with an increase in pro-inflammatory cytokines and the number of inflammatory cells, such as macrophages and neutrophils—evidence of histological tissue damage [66–68]. Although the highest concentration of oxygen as F_I_O_2_ 1.0 is definitely harmful, an F_I_O_2_ higher than 0.6 instead of 1.0 can cause various degrees of lung injury in animal models [68]. A dose- and time-dependent inflammatory pulmonary response was found in mice exposed to several degrees of hyperoxia [69]. The NO pathway, through which increasing NO can be produced in the presence of inducible-NO synthase (i-NOS) in epithelial, endothelial and immune cells, appears to play a key role in the development of pulmonary inflammation. Exposure to hyperoxia by enhancing oxidative stress may also induce the important production of cytokines and ROS [69, 70], such as peroxynitrite, a cytotoxic oxygen free radical generated from the reaction between superoxide anion and NO (Fig. 5). Hyperoxia-induced lung injury was also shown in i-NOS knockout mice compared to wild-type mice [71], indicating that other mechanisms of lung injury aside from NO production by i-NOS exist. Hyperoxia in the lungs can induce both cell death by modulating the expression of specific genes regulating the cellular survival or death [72] and by cell apoptosis, mediated by caspase families [73]. The inhalation of NO as a therapeutic strategy for hyperoxic lung injury is still controversial [74]. The surfactant system may also be impaired by hyperoxia with the downregulation of surfactant-associated protein [72, 75], alveolar instability and a reduction in lung compliance, especially during ventilation with high tidal volumes [76, 77].

In addition to the lung injury caused by oxidative stress, excessive oxygen supply impairs the antimicrobial capacity of immune cells as well as the mucociliary clearance, and hyperoxia was reported to result in aggravated lung injury and increased mortality in animal models of pulmonary infection [78–81]. The number of days suffering from hyperoxemia was also shown to be an independent risk factor for ventilator-associated pneumonia [82].

### Effects on the heart

Hyperoxia has been reported to be associated with both increased vascular resistance in the coronary artery, leading to reductions in coronary blood flow [83], and increased myocardial oxygen consumption [84]. Nevertheless, supplemental oxygen has been routinely used for more than a century in the management of acute coronary syndrome, with the rationale of increasing oxygen supply to the ischemic myocardium. Increasing evidence has shown that hyperoxemia may induce a reduction in coronary blood flow [83, 84] with a paradoxical increase in myocardial ischemia [85] and aggravated myocardial ischemia reperfusion injury [86].

In patients with cardiac arrest, oxygen therapy at a high concentration is often used during cardiopulmonary resuscitation and in the post-resuscitation period to increase oxygen availability at the tissue level. However, the potential detrimental effects of hyperoxemia and ROS toxicity have been demonstrated in cases of ischemia reperfusion syndrome [87]. In animal models, the administration of 100% oxygen after resuscitation from cardiac arrest led to worse neurological outcomes than the administration of lower oxygen concentrations [88]. A recent prospective multicenter cohort study showed that early exposure to hyperoxia of more than 300 mmHg of PaO_2_ within 6 h after resuscitation from cardiac arrest was independently associated with a poor neurological function at hospital discharge [89]. Furthermore, a meta-analysis including 16 observational studies with adult post-cardiac arrest patients showed that intra-arrest hyperoxia was associated with a lower mortality rate, while post-arrest hyperoxia was associated with a higher mortality rate [90].

### Effects on the brain

Regarding the effects on the brain, promising results were shown in preclinical studies with oxygen therapy in stroke, demonstrating a reduction in infarct size, slower damage to the blood brain barrier and a decreased risk for secondary hemorrhaging after thrombolysis [91]. However, clinical studies have failed to demonstrate any protective effect of oxygen therapy in patients with cerebrovascular pathological events. A relationship between hyperoxemia and adverse outcomes was observed in patients with stroke [92] and subarachnoid hemorrhaging [93], with a higher PaO_2_ found to be associated with a greater risk of delayed cerebral ischemia. A meta-analysis showed a minor trend toward benefits with oxygen therapy in patients with stroke. However, controversy persists since various results have been obtained in different studies such as an improvement of the short-term neurological score and a worsening of both the physical function and mortality after more than 3 months [94].

Cobley et al. suggested reasons why the brain is susceptible to oxidative stress, including unsaturated lipid enrichment, glucose, mitochondria, calcium, glutamate, modest antioxidant defense, redox active transition metals, neurotransmitter auto-oxidation, and RNA oxidation. The brain may continuously control chemically diverse reactive species in order to perform heterogeneous signaling functions [95].

### Efficacy in cases of sepsis and septic shock

Excessive supplemental oxygen during sepsis treatment may aggravate both oxidative stress and the inflammatory response, leading to a worsening organ function [56], as shown in an animal model. The results of preclinical studies remain controversial, although the use of 100% oxygen was positively proposed by several authors for its potential benefits [64, 65, 96]. In an ovine model of septic shock, hyperoxia was found to improve the hemodynamics and peripheral microvascular flow and to preserve the cerebral metabolism, renal function, and gas exchange [65]. Another similar study described the effects of hyperoxia on improving the organ function and attenuating tissue apoptosis without affecting the lung function and oxidative or nitrosative stress [64]. However, a recently published multicenter randomized control trial that evaluated the effects of hyperoxemia during the first 24 h and hypertonic saline infusion in patients with septic shock was prematurely stopped after 442 patients were recruited and assigned to a treatment group, as it was found that hyperoxemia was associated with an increased mortality (43% vs. 35%) and serious adverse events, such as ICU-acquired weakness and atelectasis [97]. In contrast, a post hoc analysis of 250 patients with sepsis enrolled in ICU-ROX55) revealed that conservative oxygen therapy did not result in a statistically significant reduction in mortality compared with usual oxygen therapy (90-day mortality: 36.2% vs. 29.2%, *p* = 0.35) [98].

## Conclusions

We should bear in mind that while supplemental oxygen can lead to a marginal increase in systemic oxygen delivery (although the degree of efficacy depends on the type of hypoxia), it may also induce serious adverse effects on inflammation, oxidative stress, the pulmonary function, microvascular perfusion, and the coronary and cerebral blood flow. Systemic and cellular oxygen-sensing systems based on the mechanisms underlying oxygen homeostasis exist to ensure a proper balance between the oxygen supply and demand. Various adaptive and maladaptive responses mediated by HIF can be evoked by hypoxia in the human body. Despite many clinical trials having been performed, no obvious evidence has yet been put forth to totally support a liberal oxygen supplementation in any subset of critically ill patients. Relatively conservative oxygen therapy with cautious monitoring appears to be safe and may improve the outcome by avoiding harmful oxidative stress resulting from excessive oxygen administration. Given the biological effects of oxygen molecules, although the optimal target levels still remain controversial, unnecessary oxygen administration should be avoided, and exposure to hyperoxemia should be minimized in critically ill patients.

## Data Availability

Not applicable

## Abbreviations

ATP: Adenosine triphosphate
ROS: Reactive oxygen species
HIF: Hypoxia-inducible factor
HAT therapy: Hydrocortisone, ascorbic acid and thiamin therapy
PO_2_: Partial pressure of oxygen
PaO_2_: Arterial PO_2_
F_I_O_2_: Fraction of inspired oxygen
COPD: Chronic obstructive pulmonary disease
NO: Nitric oxide
i-NOS: Inducible-NO synthase
ICU: Intensive care unit

## References

[1] Bitterman H (2009). Bench-to-bedside review: oxygen as a drug. Crit Care.

[2] West JB, Luks AB (2017). West’s pulmonary pathophysiology: the essentials 9th ed.

[3] Hall J (2016). Guyton and Hall Textbook of Medical Physiology.

[4] Samanta D, Prabhakar NR, Semenza GL (2017). Systems biology of oxygen homeostasis. Wiley Interdiscip Rev Syst Biol Med.

[5] Semenza GL (2010). Oxygen homeostasis. Wiley Interdiscip Rev Syst Biol Med.

[6] Wang GL, Jiang BH, Semanza GL (1995). Hypoxia-inducible factor 1 is a basic-helix-loop-helix-PAS heterodimer regulated by cellular O_2_ tension. Proc Natl Acad Sci U S A.

[7] Prabhakar NR, Semanza GL (2012). Adaptive and maladaptive cardiorespiratory responses to continuous and intermittent hypoxia mediated by hypoxia-inducible factors 1 and 2. Physiol Rev.

[8] Prabhakar NR, Semenza GL (2015). Oxygen sensing and homeostasis. Physiology (Bethesda).

[9] Semenza GL, Prabhakar NR (2018). The role of hypoxia-inducible factors in carotid body (patho) physiology. J Physiol.

[10] Iturriaga R, Oyarce MP, Dias ACR (2017). Role of carotid body in intermittent hypoxia-related hypertension. Curr Hypertens Rep.

[11] Semenza GL, Prabhakar NR (2012). The role of hypoxia-inducible factors in oxygen sensing by the carotid body. Adv Exp Med Biol.

[12] Ince C, Mik EG (2016). Microcirculatory and mitochondrial hypoxia in sepsis, shock, and resuscitation. J Appl Physiol (1985).

[13] Shoemaker WC, Appel PL, Kram HB (1993). Temporal hemodynamic and oxygen transport patterns in medical patients. Septic shock. Chest..

[14] Ince C, Ashruf JF, Avontuur JA (1993). Heterogeneity of the hypoxic state in rat heart is determined at capillary level. Am J Phys.

[15] Nelson LD (1999). Dysoxia and "dat" oxia: where does the oxygen go?. Crit Care Med.

[16] Robin ED (1977). Special report: dysoxia. Abnormal tissue oxygen utilization. Arch Intern Med.

[17] Creery D, Fraser DD (2002). Tissue dysoxia in sepsis: getting to know the mitochondrion. Crit Care Med.

[18] Ince C (2005). The microcirculation is the motor of sepsis. Crit Care.

[19] Brooks GA (2009). Cell-cell and intracellular lactate shuttles. J Physiol.

[20] Brooks GA (2000). Intra- and extra-cellular lactate shuttles. Med Sci Sports Exerc.

[21] Brooks GA (2018). The science and translation of lactate shuttle theory. Cell Metab.

[22] Gattinoni L, Vasques F, Camporota L (2019). Understanding lactatemia in human sepsis. Potential impact for early management. Am J Respir Crit Care Med.

[23] Suetrong B, Walley KR (2016). Lactic acidosis in sepsis: it's not all anaerobic: Implications for diagnosis and management. Chest..

[24] Morelli A, Ertmer C, Westphal M (2013). Effect of heart rate control with esmolol on hemodynamic and clinical outcomes in patients with septic shock: a randomized clinical trial. JAMA..

[25] Levy B, Gibot S, Franck P (2005). Relation between muscle Na^+^ K^+^ ATPase activity and raised lactate concentrations in septic shock: a prospective study. Lancet.

[26] James JH, Fang CH, Schrantz SJ (1996). Linkage of aerobic glycolysis to sodium-potassium transport in rat skeletal muscle. Implications for increased muscle lactate production in sepsis. J Clin Invest.

[27] James JH, Wagner KR, King JK (1999). Stimulation of both aerobic glycolysis and Na^+^-K^+^-ATPase activity in skeletal muscle by epinephrine or amylin. Am J Phys.

[28] Marik PE, Khangoora V, Rivera R (2017). Hydrocortisone, vitamin C, and thiamine for the treatment of severe sepsis and septic shock: a retrospective before-after study. Chest.

[29] Chang P, Liao Y, Guan J (2020). Combined treatment with hydrocortisone, vitamin C, and thiamine for sepsis and septic shock: a randomized controlled trial. Chest..

[30] Litwak JJ, Cho N, Nguyen HB (2019). Vitamin C, hydrocortisone, and thiamine for the treatment of severe sepsis and septic shock: a retrospective analysis of real-world application. J Clin Med.

[31] Damiani E, Donati A, Girardis M (2018). Oxygen in the critically ill: friend or foe?. Curr Opin Anaesthesiol.

[32] Damiani E, Adrario E, Girardis M (2014). Arterial hyperoxia and mortality in critically ill patients: a systematic review and meta-analysis. Crit Care.

[33] Helmerhorst HJ, Roos-Blom MJ, van Westerloo DJ (2015). Association between arterial hyperoxia and outcome in subsets of critical illness: a systematic review, meta-analysis, and meta-regression of cohort studies. Crit Care Med.

[34] Turrens JF (2003). Mitochondrial formation of reactive oxygen species. J Physiol.

[35] Gore A, Muralidhar M, Espey MG (2010). Hyperoxia sensing: from molecular mechanisms to significance in disease. J Immunotoxicol.

[36] Kilgannon JH, Jones AE, Parrillo JE (2011). Emergency medicine shock research network (EMShockNet) investigators. Relationship between supranormal oxygen tension and outcome after resuscitation from cardiac arrest. Circulation..

[37] Janz DR, Hollenbeck RD, Pollock JS (2012). Hyperoxia is associated with increased mortality in patients treated with mild therapeutic hypothermia after sudden cardiac arrest. Crit Care Med.

[38] Helmerhorst HJ, Arts DL, Schultz MJ (2017). Metrics of arterial hyperoxia and associated outcomes in critical care. Crit Care Med.

[39] Thomson L, Paton J (2014). Oxygen toxicity. Paediatr Respir Rev.

[40] Jenkinson SG (1988). Oxygen toxicity. J Intensive Care Med.

[41] Nishimura M (2015). High-flow nasal cannula oxygen therapy in adults. J Intensive Care.

[42] Nishimura M (2016). High-flow nasal cannula oxygen therapy in adults: Physiological benefits, indication, clinical benefits, and adverse effects. Respir Care.

[43] Nathan C (2003). Immunology: oxygen and the inflammatory cell. Nature..

[44] Brill SE, Wedzicha JA (2014). Oxygen therapy in acute exacerbations of chronic obstructive pulmonary disease. Int J Chron Obstruct Pulmon Dis.

[45] Aboab J, Jonson B, Kouatchet A (2006). Effect of inspired oxygen fraction on alveolar derecruitment in acute respiratory distress syndrome. Intensive Care Med.

[46] Magnusson L, Spahn DR (2003). New concepts of atelectasis during general anaesthesia. Br J Anaesth.

[47] Edmark L, Kostova-Aherdan K, Enlund M (2003). Optimal oxygen concentration during induction of general anesthesia. Anesthesiology..

[48] Benoît Z, Wicky S, Fischer JF (2002). The effect of increased F_I_O_2_ before tracheal extubation on postoperative atelectasis. Anesth Analg.

[49] Pacher P, Beckman JS, Liaudet L (2007). Nitric oxide and peroxynitrite in health and disease. Physiol Rev.

[50] Wright CJ, Dennery PA (2009). Manipulation of gene expression by oxygen: a primer from bedside to bench. Pediatr Res.

[51] Davies KJ (1995). Oxidative stress: the paradox of aerobic life. Biochem Soc Symp.

[52] Oyewole AO, Birch-Machin MA (2015). Mitochondria-targeted antioxidants. FASEB J.

[53] Helmerhorst HJ, Schultz MJ, van der Voort PH (2015). Bench-to-bedside review: the effects of hyperoxia during critical illness. Crit Care.

[54] Mackle D, Bellomo R, Bailey M (2020). ICU-ROX Investigators the Australian and New Zealand Intensive Care Society Clinical Trials Group. Conservative oxygen therapy during mechanical ventilation in the ICU. N Engl J Med.

[55] Barrot L, Asfar P, Mauny F (2020). LOCO_2_ Investigators and REVA Research Network. Liberal or conservative oxygen therapy for acute respiratory distress syndrome. N Engl J Med.

[56] Rodríguez-González R, Martín-Barrasa JL, Ramos-Nuez Á (2014). Multiple system organ response induced by hyperoxia in a clinically relevant animal model of sepsis. Shock..

[57] Thomson AJ, Drummond GB, Waring WS (2006). Effects of short-term isocapnic hyperoxia and hypoxia on cardiovascular function. J Appl Physiol (1985).

[58] Modun D, Krnic M, Vukovic J (2012). Plasma nitrite concentration decreases after hyperoxia-induced oxidative stress in healthy humans. Clin Physiol Funct Imaging.

[59] Attaye I, Smulders YM, de Waard MC (2017). The effects of hyperoxia on microvascular endothelial cell proliferation and production of vaso-active substances. Intensive Care Med Exp.

[60] Orbegozo Cortés D, Puflea F, Donadello K (2015). Normobaric hyperoxia alters the microcirculation in healthy volunteers. Microvasc Res.

[61] Milstein DM, Helmers R, Hackmann S (2016). Sublingual microvascular perfusion is altered during normobaric and hyperbaric hyperoxia. Microvasc Res.

[62] Donati A, Damiani E, Zuccari S (2017). Effects of short-term hyperoxia on erythropoietin levels and microcirculation in critically ill patients: a prospective observational pilot study. BMC Anesthesiol.

[63] Rossi P, Tauzin L, Weiss M (2007). Could hyperoxic ventilation impair oxygen delivery in septic patients?. Clin Physiol Funct Imaging.

[64] Barth E, Bassi G, Maybauer DM (2008). Effects of ventilation with 100% oxygen during early hyperdynamic porcine fecal peritonitis. Crit Care Med.

[65] He X, Su F, Xie K (2017). Should Hyperoxia Be Avoided During Sepsis? An Experimental Study in Ovine Peritonitis. Crit Care Med.

[66] Nagato AC, Bezerra FS, Lanzetti M (2012). Time course of inflammation, oxidative stress and tissue damage induced by hyperoxia in mouse lungs. Int J Exp Pathol.

[67] Altemeier WA, Sinclair SE (2007). Hyperoxia in the intensive care unit: why more is not always better. Curr Opin Crit Care.

[68] Kallet RH, Matthay MA (2013). Hyperoxic acute lung injury. Respir Care.

[69] Helmerhorst HJF, Schouten LRA, Wagenaar GTM (2017). Hyperoxia provokes a time- and dose-dependent inflammatory response in mechanically ventilated mice, irrespective of tidal volumes. Intensive Care Med Exp.

[70] Bhandari V, Elias JA (2006). Cytokines in tolerance to hyperoxia-induced injury in the developing and adult lung. Free Radic Biol Med.

[71] Hesse AK, Dörger M, Kupatt C (2004). Proinflammatory role of inducible nitric oxide synthase in acute hyperoxic lung injury. Respir Res.

[72] Shimada I, Kubota A, Katoh M (2016). Hyperoxia causes diffuse alveolar damage through mechanisms involving upregulation of c-Myc/Bax and enhanced production of reactive oxygen species. Respir Investig.

[73] Makena PS, Luellen CL, Balazs L (2010). Preexposure to hyperoxia causes increased lung injury and epithelial apoptosis in mice ventilated with high tidal volumes. Am J Phys Lung Cell Mol Phys.

[74] Liu WW, Han CH, Zhang PX (2016). Nitric oxide and hyperoxic acute lung injury. Med Gas Res.

[75] Schwingshackl A, Lopez B, Teng B (2017). Hyperoxia treatment of TREK-1/TREK-2/TRAAK-deficient mice is associated with a reduction in surfactant proteins. Am J Phys Lung Cell Mol Phys.

[76] Bailey TC, Martin EL, Zhao L (2003). High oxygen concentrations predispose mouse lungs to the deleterious effects of high stretch ventilation. J Appl Physiol (1985).

[77] Sinclair SE, Altemeier WA, Matute-Bello G (2004). Augmented lung injury due to interaction between hyperoxia and mechanical ventilation. Crit Care Med.

[78] Tateda K, Deng JC, Moore TA (2003). Hyperoxia mediates acute lung injury and increased lethality in murine Legionella pneumonia: the role of apoptosis. J Immunol.

[79] Kikuchi Y, Tateda K, Fuse ET (2009). Hyperoxia exaggerates bacterial dissemination and lethality in Pseudomonas aeruginosa pneumonia. Pulm Pharmacol Ther.

[80] Saito K, Kimura S, Saga T (2013). Protective effect of procysteine on Acinetobacter pneumonia in hyperoxic conditions. J Antimicrob Chemother.

[81] Baleeiro CE, Wilcoxen SE, Morris SB (2003). Sublethal hyperoxia impairs pulmonary innate immunity. J Immunol.

[82] Six S, Jaffal K, Ledoux G (2016). Hyperoxemia as a risk factor for ventilator-associated pneumonia. Crit Care.

[83] McNulty PH, King N, Scott S (2005). Effects of supplemental oxygen administration on coronary blood flow in patients undergoing cardiac catheterization. Am J Physiol Heart Circ Physiol.

[84] Farquhar H, Weatherall M, Wijesinghe M (2009). Systematic review of studies of the effect of hyperoxia on coronary blood flow. Am Heart J.

[85] Guensch DP, Fischer K, Shie N (2015). Hyperoxia exacerbates myocardial ischemia in the presence of acute coronary artery stenosis in swine. Circ Cardiovasc Interv.

[86] Muntean DM, Sturza A, Dănilă MD (2016). The role of mitochondrial reactive oxygen species in cardiovascular injury and protective strategiesx. Oxidative Med Cell Longev.

[87] Llitjos JF, Mira JP, Duranteau J (2016). Hyperoxia toxicity after cardiac arrest: What is the evidence?. Ann Intensive Care.

[88] Pilcher J, Weatherall M, Shirtcliffe P (2012). The effect of hyperoxia following cardiac arrest - A systematic review and meta-analysis of animal trials. Resuscitation..

[89] Roberts BW, Kilgannon JH, Hunter BR (2018). Association between early hyperoxia exposure after resuscitation from cardiac arrest and neurological disability: Prospective multicenter protocol-directed cohort study. Circulation..

[90] Patel JK, Kataya A, Parikh PB (2018). Association between intra- and post-arrest hyperoxia on mortality in adults with cardiac arrest: A systematic review and meta-analysis. Resuscitation..

[91] Shi SH, Qi ZF, Luo YM (2016). Normobaric oxygen treatment in acute ischemic stroke: a clinical perspective. Med Gas Res.

[92] Rincon F, Kang J, Maltenfort M (2014). Association between hyperoxia and mortality after stroke: a multicenter cohort study. Crit Care Med.

[93] Jeon SB, Choi HA, Badjatia N (2014). Hyperoxia may be related to delayed cerebral ischemia and poor outcome after subarachnoid haemorrhage. J Neurol Neurosurg Psychiatry.

[94] Ding J, Zhou D, Sui M (2018). The effect of normobaric oxygen in patients with acute stroke: a systematic review and meta-analysis. Neurol Res.

[95] Cobley JN, Fiorello ML, Bailey DM (2018). 13 reasons why the brain is susceptible to oxidative stress. Redox Biol.

[96] Calzia E, Asfer P, Hauser B (2010). Hyperoxia may be beneficial. Crit Care Med.

[97] Asfer P, Schortgen F, Boisramé-Helms J (2017). Hyperoxia and hypertonic saline in patients with septic shock (HYPERS2S): a two-by-two factorial, multicentre, randomised, clinical trial. Lancet Respir Med.

[98] Young P, Mackle D, Bellomo R, ICU-ROX Investigators the Australian New Zealand Intensive Care Society Clinical Trials Group (2020). Conservative oxygen therapy for mechanically ventilated adults with sepsis: a post hoc analysis of data from the intensive care unit randomized trial comparing two approaches to oxygen therapy (ICU-ROX). Intensive Care Med.

